# On the Constitutional Effects of Anæsthetic Agents

**Published:** 1856-11

**Authors:** J. Henry Clark

**Affiliations:** Newark, N. J.


*On the Constitutional Effects of Anæsthetic Agents.*—By J. Henry Clark, M.D., of Newark, N. J.

New remedies, like new systems of medicine, are usually inaugurated with extravagant promises. Zealous advocates adopt and publish the most flattering encomiums upon the last Eureka, which, unlike its predecessors, is quite certain never to sink below the high elevation upon which they have placed it. Little observation is necessary to note how speedily the experience of practical men determine the real position of all these newly discovered dogmas and remedies. Many remedies most highly praised never find a permanent place in the estimation of the practitioner, and very many so-called systems which are ushered in with great eclat, which secure much popular favor, and find many advocates, leave scarce a single contribution to the treasury of science, or add the smallest stone to that temple which we are ever striving to rear. A volume could be composed of new discoveries, each of which, in their day, furnished subjects for the journals, medical and secular, the very names of which have long since dropped from memory and record. It is scarcely necessary to refer to stramonium that was to cure all epilepsies, the remedy for hydrophobia; the styptic, that was to control all hæmorrhages; the digitalis, that was to control the heart; and the ergot, which was certain to relieve most of the difficulties of the lying-in chamber. These and a host of others have taken useful places in the list of appliances, by means of which the physician may often control disease, usually fulfilling one or two, instead of many, medications. In the same manner many of the "isms," "pathy," and "specialities," after making fortunes for quacks, have their day with the public, (who forget all about them, their attention being devoted to a new one just rising,) and at length become sifted, and the truth, often the merest kernel, is added to the list of tested and reliable facts.

One of the latest discoveries that has challenged investigation, that has furnished the subject of many a newspaper paragraph, and many a chapter for our medical journals, is that of chloroform. It was introduced as one of the discoveries that would immortalize its originators. They have been rewarded with high honors and Government appropriations. The new discovery has furnished an illustration of progress and development for the orator and the author, who have regarded it as the panacea for physical suffering, even that which was entailed upon our race, in the curse pronounced upon its maternal progenitor. It was resorted to freely if a tooth was to be extracted, or a toe-nail removed. Surgeons were recommended to use it in even trifling operations, and it is now declared in high places to afford a safe and desirable means of relieving the pains of parturition. As might have been expected, an agent so potent as, by a breath, to rob the patient of his senses, sometimes was found to produce the most mischievous effects. Patients died under its use. The partisans of the new remedy told us that the "cases were not well selected;" that "the article was impure;" that, if properly employed, it was entirely safe. With regard to the selection of the cases, these writers said little, but commonly referred to their own experience, if they had chanced to be successful.

It seems to have become established that there is no rule in relation to the condition of the patient, or the purity of the article, by means of which it can be employed with any degree of security. It seems to be fast settling down into the place of affording valuable assistance in emergencies, and during the performance of capital operations. That it is a very valuable remedy, as a soporific, when diffused in the atmosphere about the patient who suffers from dyspnœa, from asthma, or any other cause, when locally applied, or when occasionally administered by the stomach, the author has almost daily evidence. That it is safe in its immediate effects, or salutary in its after influence, when introduced into the lungs in such quantities as to produce insensibility, he does not believe. In view of the casualties that are frequently reported (less frequently as the article is becoming less used,) few physicians would be willing to breathe these agents themselves, in order to experiment upon their effects. The author has observed it produce the most strange hallucinations, which continued to occupy the mind and make upon it the impression of reality, long after sanity and health was restored. He has found no difficulty in believing in the possibility of the entire innocence of Beale, the imprisoned dentist, in Philadelphia; having seen cases of hallucination in his practice, far more remarkable and improbable.

Enough has been written of its *immediate* effects. It was noteworthy that, at the meeting of dentists held, not very long since, in this city, most declared their unwillingness to use it frequently; many of them furnished cases illustrative of the peculiar, strange, and fatal symptoms which sometimes followed its administration; but nowhere have I observed anything in relation to the *permanent influence* upon the constitution produced by the inhalation of anæsthetic agents, several cases having fallen under my observation, which has induced me to regard the whole danger as not entirely passed when the inhalation has been effected without exhibiting any phenomena indicating danger.

*Case* 1.—Mr. J. M., a gentleman, about 30 years of age, of good constitution, who ordinarily enjoyed good health, took *ether* to facilitate the extraction of a tooth. He is of a nervous temperament, light complexion, blue eyes, and light hair. The administration of the agent produced no unusual effect, except that a "choking feeling" was experienced just before the occurrence of complete insensibility. After recovery from the immediate effect, for several weeks, extreme nausea and constant pain in the head was experienced.

After the lapse of three or four months, Mr. M. applied to me for advice, complaining of the following train of symptoms, the commencement of which he dated from the day that he inhaled the ether. He is an intelligent man, and has no doubt that they are wholly attributable to the inhalation, and that they commenced at that time. These symptoms, he says, he never before experienced. Is very "bilious" (to use his own language); tongue much coated; constant pain in the head, especially over the eyes; a "feeling as if there was a *want of mental exercise*;" habitual costiveness; pain in the right side and back; indigestion, want of muscular strength; nervous system greatly deranged; neuralgic pains; and great despondency of mind.

Change of habits, mineral tonics and alteratives, restored him to perfect health after a few months. All these symptoms at length disappeared.

*Case* 2.—Mr. C. M. inhaled *chloroform* for the purpose of having a tooth extracted in November, 1854. In the afternoon of January 5,

1855, I was called hastily, found him in a state of dreadful nervous excitement. He had just recovered from a period of insensibility; his extremities were cool; pupils of the eyes natural; some heat about the head and neck; convulsive twitchings in the face; and trembling with nervous agitation and mental excitation.

When the urgent symptoms were relieved, I learned that he recovered from the immediate effects of the inhalation without any unusual symptom; that it was followed by a very unpleasant feeling in the top of the head, such as he had never experienced before; that this symptom and neuralgic pains had been constantly experienced from the day that he took the chloroform.

In the morning of January 6th, while lying reading on the sofa, he was seized with a singular sensation of bewilderment and other feelings that he finds no language to describe. These sensations greatly alarmed him. Recovering himself, he went to his father's warehouse, while there engaged, an hour or two afterwards, he again experienced those singular sensations, but found himself unable to *articulate distinctly*. His father perceiving that he was ill, observing convulsive twitching in his face, with palor and anxiety, and perceiving in his conduct a desire to go home, took his arm and walked with him to his residence, about half a mile distant. I was soon after summoned, and found him in the condition heretofore described.

I prescribed stimulants, antispasmodics, and alteratives. He did not leave his room for a week, during which he suffered from extreme restlessness, anxiety and nertigo. His pulse continued, during the week, depressed. I treated him with tonics and antispasmodics and mild mercurial alteratives with evident advansage; but his neuralgic symptoms yielded slowly. His liver was constantly torpid; lassitude, indecision, and depression of spirits were indicated in his conduct.

I advised a voyage to Europe. He sailed in May, and remained till September. He returned very much benefitted, but had the same furred tongue that was evident in the case previously referred to, with more or less habitual costiveness. These symptoms have yielded to the use of mild alteratives, and he seems now, after the lapse of fifteen months since its inhalation, to have recovered again his wonted strength and vigor.

In the case last alluded to, it required about eighteen months to rally from the influence of the ethereal inhalation. In the case to which I shall next refer, over two years was required to outlive the same train of symptoms, mostly neuralgic, which commenced from the day of the inhalation.

*Case* 3.—Mrs. W., about 25 years of age, dark complexion, dark eyes, dark hair, of sanguine temperament, none of the nervous. She had usually enjoyed good health, although not robust. She received *chloroform* at the hands of a dentist also. I was called a year afterwards to prescribe for a set of symptoms very much like those described in the cases before narrated; neuralgia and "strange feelings about the head," were the prominent symptoms described, with a general feeling of "malaise." I found, as in the other cases, a determined conviction that all these symptoms were new, and that they commenced with the inhalation. I treated her as I did the others, depending mainly upon *iron;* the appearance of the countenance indicated the want of a larger supply of iron in the blood. She gradually regained her usual health. It required a full year, however, to accomplish the cure. The writer believes that the experience of others, if it could be accumulated, would prove that—

1. Chloroform is most dangerous when employed in cases of trifling importance, both in relation to its immediate effects and final results. This is proved by the greater frequency of cases in which unpleasant results are observed, which follow the extraction of teeth or its use in trifling operations, where there is not pain enough to resist its excessive effects.

2. That persons whose nervous systems are particularly susceptible are most liable to suffer from the inhalation of these agents.

3. That the use of chloroform in the lying-in chamber is not devoid of danger.

A very limited inquiry into the experience of others has been made with the following results. An eminent practitioner, in answer to my inquiry, says: "I have given it in four cases of accouchment. One of my patients had puerperal mania, and another congestion of the brain, both within six days afterwards. It is possible that chloroform was not the cause in either case, but I have never ventured to use it since. A lady of eminence in New Jersey, took choloroform in labor, became comatose, immediately recovered, with *permanently impaired* intellect.

In the experience of a neighboring practitioner, a perfectly healthy girl aged 18, sanguine temperament—catamenia regular, applied to a dentist to have a tooth extracted under the influence of chloroform. It was done very carefully by a prudent man. A very few inspirations sufficed to produce insensibility. The tooth was extracted, but sensibility did not return for several days—a serious congestion of the brain followed, continuing for several weeks, and though several years have elapsed, she continues in a partially demented condition, subject to periods of excitement somewhat like those which she exhibited during the original attack.

A physician of New York City relates a case of a young woman aged 28, in good health, who, after the extraction of a tooth, was immediately seized with the most distressing symptoms. Every muscle in the body commenced jerking and twitching. These phenomena continued for four days, despite the means adopted for her relief. They ceased at length under the use of strychnine. I am not informed with regard to the influence upon the mind.

The same gentleman related the case of a young girl, in a good family, (without date, age, name or any facts lest the matter might become public, and grieve the friends,) who became crazed immediately after taking chloroform to facilitate the extraction of a tooth—*years have passed, and she is still a maniac.*

Another case has come to my knowledge of a child fourteen years old, who took chloroform when about to undergo a trifling operation; she became deranged; *six years have passed, and she has in nowise recovered.*

Morris County, N. J., furnishes a case, that I am not at liberty to report, in which permanent idiocy resulted from the inhalation of chloroform.

That these agents should be used with great caution, is further proved from their influence upon certain susceptible constitutions, when not taken for the purpose of producing insensibility, but when merely exposed to an atmosphere charged with the fumes of ether or chloroform. On this point I have several illustrations, but one will suffice.

Mr. ———, a highly respectable dentist of ———, of nervous habit, light complexion, active motion, suffered from all the symptoms which were observable in the cases which I reported in my own experience in the early part of this article, simply from breathing the fumes of these anæsthetic agents as they permeated the apartments in which he operated and resided. He was accustomed to use these agents in his practice, almost hourly; his house was perpetually filled with the odor. He became, at length, feeble, pulseless, anemic—"felt as if he had been drinking champaigne"—suffered from neuralgia, and very great nervous debility. Abandonment of business, a residence in the country for three years and constant out-door exercise, and the use of electricity, accomplished a cure at the *end of that period.* He has no doubt but his illness resulted from this cause, and that his recovery is due to its removal, and the remedies employed.

My honored and beloved preceptor, the late James C. Bliss, M. D., related a case to me, last Spring, in which amaurosis followed by idiocy, resulted from the use of chloroform or ether in parturition. They succeeded the inhalation so promptly that it is evidently proper thus to trace cause and effect. I understood that all who saw her, both in and out of the profession, attributed the amaurosis and the subsequent derangement to the inhalation of one of these agents; I did not understand which.

Several other cases have come to my notice, in which the inhalation of anæsthetics seemed to produce similar constitutional effects. They were not my own patients, and I could not get access to well-authenticated facts in relation to them.

These facts have made upon my own mind the impression that these agents should be regarded as remedies of great potency, and not always certain to produce none but salutary effects; that they should not, if possible, be administered to persons, subject to local determination, or where their antecedents offer reason to apprehend derangement; that their use should be avoided in persons of extremely nervous temperament, or for any cause of nervous susceptibility.

We have the authority of eminent men in Europe, and in this country, whose experience in the use of these agents has been very extensive, in favor of their usefulness and safety. Still these gentlemen have committed themselves in favor of what may be regarded as their hobby, without being chargeable with any want of respect, and the experience of others does not justify fully their opinions with regard to their entire safety.

If cases of dying do *sometimes* occur in the lying-in chamber, and on the operating tables—if debility, hepatic congestion, neuralgia, apoplexy, and mania, do *sometimes* follow their use—it would appear proper that he who would deserve a reputation for wisdom and discretion should be quite certain that the severity of the case demands a *somewhat hazardous* remedy. Chloroform furnishes most valuable assistance in the operations of surgery, and the lying-in chamber. With no disposition to undervalue it, and far less to treat lightly the valuable statistical evidence furnished by Professor Channing, of Boston, and Professor Simpson, of Edinburgh, of its comparative safety, when carefully employed, I cannot believe that its employment to relieve the pangs of *ordinary confinement*, or to allay the pain of a trifling operation, is wise or expedient. If, in my comparatively limited experience, three cases should come under my observation within about two years, others, situated more advantageously, could note many such cases, if looked for. To record the result of my observations, and to prompt abler observers to look in the same direction, is the whole of my present purpose.

While writing the concluding pages of this article, I have been called to administer chloric ether in a case of amputation of the adventitious toe of each foot, from a young gentleman of vigorous health. Although the operation was not very protracted, and the ether was administered by a discreet medical assistant, derangement followed; nor were my anxieties entirely relieved with regard to these effects before the lapse of six or eight hours.

If the facts which I have collated are sustained by those of larger experience—if there are constitutional effects, as well as immediate, to be guarded against, more regard should be given to the habits of the patient, and these agents should surely be reserved for grave and trying emergencies.

That permanent ill effects should be produced by agents that cause insensibility or derangement, is to be expected by analogy, and by previous experience with regard to other agents which produced like phenomena. I have not access to the facts in the case, but, if I mistake not, the same kind of effects, both immediate and constitutional, were experienced in the case of Professor Fisk by passing a current of electricity through the diaphragm. If the reader has access to the facts he will find it a case in point.

In an early volume of the *Amer. Jour. of Science & Arts* is an account of the constitutional effect of the inhalation of the "Nitrous Oxide Gas." The subject of the experiment, immediately after taking the exhilarating gas, began to laugh immoderately, and laughed incessantly for years afterwards. It is furthermore related, if I remember correctly, that recovery never took place.

It is likely that we shall never discover a perfectly safe and harmless way of parting company with our senses, with the expectation that, after the object is accomplished, be it but the advance of science, or the relief of pain, we can be quite certain that reason and intellect, will promptly return.—*New York Journal of Medicine.*

